# Estimate of Occupational Exposure to Carcinogens among Migrant Workers in the United Arab Emirates: A Cross-Sectional Study

**DOI:** 10.3390/ijerph192013012

**Published:** 2022-10-11

**Authors:** Iffat Elbarazi, Sonia El-Zaemey, Basema Saddik, Balázs Ádám, Mohamed El Sadig, Aminu S. Abdullahi, Lin Fritschi, Mohamud Sheek-Hussein

**Affiliations:** 1 Institute of Public Health, College of Medicine and Health Sciences, United Arab Emirates University, Al Ain 15551, United Arab Emirates; 2 School of Population Health, Curtin University, Bentley 6102, Australia; 3 Department of Family and Community Medicine and Behavioral Sciences, College of Medicine, University of Sharjah, Sharjah 27272, United Arab Emirates; 4 Sharjah Institute of Medical Research, University of Sharjah, Sharjah 27272, United Arab Emirates

**Keywords:** carcinogenic exposure, prevalence, cross-sectional survey, UAE, OccIDEAS, occupational cancer, self-rated health

## Abstract

Occupational illnesses, such as cancer, cause more deaths each year than occupational accidents. Occupational carcinogens include physical, chemical, biological and organizational hazards. In the United Arab Emirates (UAE), migrant workers account for 80% of labor. Being sometimes employed as unskilled workers and more willing to work in demanding jobs, their vulnerability and exposure may be increased. This study aimed to estimate the prevalence of occupational exposure to workplace carcinogens among migrant workers in the UAE. A sample of employees working in construction, cleaning, dry cleaning, mechanic workshops and hair salons were recruited and interviewed. Using OccIDEAS (an online assessment tool), participants were asked questions about their demographics, work history and regular tasks. Exposure to various carcinogens was estimated using the in-built algorithms of OccIDEAS. A sample of 1778 workers was included. The sample consisted of workers from Bangladesh (19.2%), India (31%), Nepal (4.7%), Pakistan (29.9%) and the Philippines (4.8%), with the rest from other nationalities. Overall, the prevalence of probable exposure was considerable, with the highest among drivers (96%) and the lowest among laundry workers (52%). Moderate to high exposure was found to 20 different carcinogens. Self-rated health among those who were exposed to carcinogens was significantly lower than among those not exposed (AOR = 0.783, 95% CI [0.638–0.961]). Exposure to several different carcinogens is relatively common in the UAE among migrant workers. Further strengthening policies and the implementation of tailored interventions are needed to prevent exposure to occupational carcinogens and, consequently, to combat occupational cancer in the UAE.

## 1. Introduction

Occupational injuries include acute conditions, such as lacerations, bruises and fractures, while occupational diseases encompass mainly chronic illnesses, such as cancer, asthma and hearing loss. Work-related cancers cause a vast and increasing disease burden worldwide [1]. Carcinogens are agents, known to cause cancer in humans, which can be classified into three major categories: chemical carcinogens (including those from biological sources), physical carcinogens and oncogenic (cancer-causing) viruses, such as HIV [2]. Exposure to these agents increases cancer incidence [3] causing a broad range of different types of malignancies [4].

Currently, it is estimated that there are 150 million people working outside their country of birth [5,6]. Migration is primarily motivated by employment and economic growth [7,8]. Due to challenging economic conditions in source countries, combined with an increasing shortage of skilled workers in destination countries, it is anticipated that the numbers of migrant people will increase rapidly in the coming years. The World Migration Report of 2020 of the International Organization for Migration (IOM) classified approximately 272 million people as international migrants in June 2019, a figure that is 51 million higher compared to the year 2010. Among these people, around two-third were migrant workers [5]. Identifying the types and numbers of carcinogens to which immigrant workers are exposed will allow targeted interventions aimed at reducing exposure, which may consequently reduce the incidence of occupational cancers [6].

Migrant workers are among the most vulnerable workers as they are often involved in the 3D jobs (Dirty, Dangerous and Demanding (or Difficult, Demeaning)) [9]. Laborers are strongly attracted to migrant work due to lack of employment in their original home countries and also due to the relatively better salaries in the host countries compared to payments in similar jobs back home [9]. Moreover, these workers tend to work for less payment and for longer hours, and often attend complex, less attractive and more risky tasks than their native-born counterparts [9]. Therefore, there is a high demand for migrant workers on the labor market, especially in jobs with a higher risk of occupational diseases and injuries [10,11,12]. A comparison between the health status of migrant workers and domestic workers is hard to make due to the lack of data. However, several studies suggest that migrant workers take more sick leave than domestic workers [8]. The reason for this could be the poor working conditions of migrant workers [8].

Considerable efforts have been made to decrease injuries at the workplace compared to the efforts made to control the risks of occupational diseases, despite the professional estimates that they cause more deaths each year than occupational accidents [13,14,15]. Occupational diseases usually result from repeated or long-term exposure to a workplace hazard, e.g., diesel exhaust or shift work. To prevent occupational diseases, it is important to estimate the number of workers exposed to hazardous agents or events and to determine the exposure sources. Several workplace hazards are poorly evaluated for their carcinogenic potential due to the rarity of primary exposure data and the complexity of establishing the epidemiologic evidence of a carcinogenic effect that is needed for quantitative analysis and proper evaluation [4]. Moreover, the analysis of workplace hazards among migrant workers is also very limited. The existing literature shows that migrant workers have greater exposure to workplace hazards compared to domestic workers, even when working within the same industry or in the same occupation [16,17].

Various studies have estimated the proportion of occupational cancer and the prevalence of exposure to occupational carcinogens in specific countries [18,19,20]. Previous studies estimated that occupational factors cause 2–8% of cancer cases [21,22,23,24,25]. Other research explored specific types and sites of occupational cancer, and investigated the contribution of specific carcinogenic agents to their development. According to the systematic review by Micallef et al., some occupational carcinogens had especially strong association (relative risk over 5) with certain cancers, such as nickel and wood dust with the development of nasal cavity cancer [26].

An Australian study estimated that 40.3% of current workers are exposed to carcinogens at workplaces and that 1.4% of all cancers reported in Australia were somehow attributable to occupational carcinogens [18,19,20,27], while previous studies have estimated a higher attributable fraction [21]. A recent study in Korea estimated the prevalence of carcinogenic workplace exposures and found welding fumes, ultraviolet radiation, ionizing radiation and mineral oils to be the most frequent hazards [28]. However, very few of these studies estimated exposures to workplace carcinogens among migrant workers. One of these studies reported that workers in Australia who completed an interview in their native language were more likely to be exposed to a carcinogen at their workplace compared to workers who gave the interview in English [29]. Moreover, the study reported that foreign-born workers were more likely to be exposed to specific carcinogens, such as silica, than Australian-born workers when working in the same job, suggesting that migrant workers may be doing the more hazardous tasks within the job [30].

Globally, Arabian Gulf countries are known to have the highest proportion of migrant workers, with 36% of their workforce consisting of migrants [9]. It has been reported that the Gulf countries are currently hosting over 10 percent of all migrants globally [9]. In some Arab countries, foreign-born workers comprise more than 80% of the working population. For instance, in 2015, the United Arab Emirates (UAE) had the fifth largest international migrant stock of workers globally with eight million migrants [31], where migrant workers accounted for 93% of the workforce [31,32].

The percentage of migrant workers in the UAE is estimated to constitute 90% of the workforce in the private sector [10]. Migrant workers in the UAE come from various countries, with the majority from South and South East Asia, i.e., Bangladesh, India, Indonesia, Pakistan, the Philippines and Sri Lanka [32]. The UAE government announced a new federal labor law (Federal Decree-Law No. 33 of 2021) regarding the regulation of labor relations effective as of February 2022, which aims to unify government and private sector working environments and facilities [33]. To the best of our knowledge, until today, no studies have estimated the exposure among these workers to carcinogenic hazards.

This research aims to investigate carcinogenic workplace exposures among employees in the UAE, with the objective of providing an insight about the prevalence and probable level of such exposures among UAE migrant workers. Currently, such information is not available; therefore, the findings of this study are crucial to assess the burden of carcinogenic exposures on immigrant workers’ health in order to understand the scale of the problem and to inform the future planning of preventive interventions aiming to control the problem.

## 2. Materials and Methods

### 2.1. Study Sample and Recruitment

This cross-sectional study was conducted in two major cities in two Emirates of the UAE (Al Ain city in the Emirate of Abu Dhabi and Sharjah city in the Emirate of Sharjah). The data collection took place between November 2020 and March 2021 in Al Ain city industrial zone that is governed by Al Ain Municipality and in the preventive medicine and visa screening centre in Sharjah city that is governed by Sharjah Municipality. We enrolled employees working in occupations that represented diverse populations regarding their occupational carcinogenic exposure and were easily accessible in Al Ain and Sharjah. Migrant workers who worked in construction, mechanical workshops, carpentry workshops, laundries, beauty and hair salons, and drivers were included in the study after consultation with authorities in both municipalities regarding their availability. We aimed to recruit 200–300 participants from each job category. Al Ain Municipality team provided us with a list of workshops and specified occupations and targeted workers’ locations, which facilitated the random recruitment process. In Sharjah, workers in specific occupations were randomly recruited in the visa screening centre during their visit for visa renewal. Inclusion criteria included consenting to participate, being a migrant worker in one of the above occupations, over 18 years old and residing in the UAE for at least one year.

The study received Institutional Research Board approval from the Social Science Research Ethics Committee of the United Arab Emirates University. Additionally, researchers obtained approval to conduct the study from Al Ain City Municipality and Sharjah Municipality.

### 2.2. Data Collection

Since most of the workers in these specific occupations in the UAE are South Asians, we employed six multilingual part-time interviewers who spoke Urdu, Hindi, Bengali, English and Arabic. A member of the research team was appointed as a project manager. All six interviewers received extensive training on the survey and the data collection from the research team based in Australia and the UAE. The Australian team visited Al Ain city in December 2019 prior to the COVID-19 pandemic and provided the UAE team, including the project manager, with training on how to use the OccIDEAS tool that was applied for data collection [34,35]. Due to the pandemic situation, the training to all other recruiters was conducted online via Zoom. After the COVID-19 pandemic restrictions eased, the research team was able to resume the data collection process. The project manager followed up with all recruiters to ensure proper guidance on the use of the tool and the accuracy of the data collection process.

The survey tool, which has been successfully used by several studies [18,34,35], contains specific questions about the job and work tasks, and uses evidence-based algorithms to assess the existence and probable level of exposure to each agent. In addition to the occupational information, collected data included demographic and lifestyle characteristics and self-rated health on a 4-item scale (‘poor’, ‘good’, ‘very good’, ‘excellent’).

The OccIDEAS web-based application was uploaded on portable electronic equipment (Samsung A tablets). All participants received verbal information about the study in their own language. After confirming their understanding of the aims and objectives of the survey and consenting to the study, participants were interviewed by the field researchers, who helped them to complete the questionnaire using instant translation.

### 2.3. Exposure Assessment

Based on their job title and main tasks, participants were assigned to one of the five job-specific modules (drivers, construction workers, dry cleaners, hairdressers and mechanics) within OccIDEAS. Each job module contained a series of questions about the tasks specific to their job that are likely to involve exposure to carcinogens, including detailed questions about how those tasks were performed, and whether any control measures (including ventilation, using new generation diesel engines (lower emission technology), working in shaded areas, respiratory equipment, gloves and other protective clothing) were used.

Based on the answers to the questions in the job module, an automatic assessment of the probability (‘none’, ‘possible’ or ‘probable’) of exposure to 38 carcinogens was provided by the built-in algorithms of OccIDEAS. For possible exposures, the project staff used all collected information to classify them into either ‘probable’ exposure or ‘none’. For workers who were assigned a probable exposure, an automatic assessment of the level (‘low’, ‘medium’, ‘high’) was also provided by the tool. The assessment rules took into account the use of exposure control measures wherever such information was available. For example, the level of exposure decreased from high to low if a worker reported using respiratory protection when using a power tool to cut wood.

### 2.4. Data Analysis

As the used tool is web-based, a special online hyperlink for the survey data was created by the OccIDEAS team to ensure accessibility. Data were downloaded from OccIDEAS into an Excel spread sheet where the database was developed and cleaned. Finally, data were analyzed using IBM Statistical Package for the Social Sciences (SPSS, version 26.0, Chicago, IL, USA).

Descriptive statistics were used to analyse the demographic and occupational characteristics of participants, and to estimate the prevalence of exposure to the various carcinogens by estimated exposure level and by job category.

Ordinal logistic regression was performed to assess the association between carcinogen exposure and self-rated health. Crude and adjusted odds ratios (COR and AOR) and their 95% confidence intervals (CI) were calculated with adjustment for age, sex, smoking and alcohol consumption. Statistical significance was accepted at 5% level.

## 3. Results

### 3.1. Demographic Characteristics

A total population of 1778 workers from the targeted occupational groups were included in the study. Around fifty-nine percent of participants were from Al Ain, Abu Dhabi Emirate, while the rest were from Sharjah Emirate. The majority of workers were young, nearly two-thirds (65%) were aged between 18 and 34 years, while only a small proportion (1.7%) were 55 years or older (Table 1). The vast majority of participants (91.8%) were males, mostly from India (31%), Pakistan (29.9%) and Bangladesh (19.2%). Nearly 80% received a monthly salary below AED 3000 (equivalent to USD 810), while half of them received a monthly salary below AED 1500 (equivalent to USD 405). Only twelve percent of the participants were educated up to graduate level, while the rest had no or only primary school education.

About one in five (19.7%) were current smokers. Alcohol consumption was not common among the participants as only about fifteen percent reported consuming alcohol (Table 1).

### 3.2. Occupational Characteristics

The majority of participants (42.7%) worked in small companies (5 to 9 workers), while only a small proportion (6.4%) worked in relatively large companies with more than 200 employees (Table 1). The respondents represented the surveyed industries with the majority (29.7%) from the construction industry, followed by salon workers (15.2%), laundry workers (14.3%), commercial drivers (14.2%), mechanics (13.9%) and cleaners (12.7%). More than half of the participants (53.2%) had been in the same job for 1–5 years, and 29.2% for 6–10 years, while 17.5% had worked more than ten years in the job.

### 3.3. Exposure to Carcinogens

The prevalence of exposure to specific carcinogens is summarized in Table 2. Overall, the prevalence rates were found to be the highest among drivers (96%) and the lowest among laundry workers (52%). One in every three participants (34.3%) was exposed to diesel exhaust, while the exposure to cadmium (0.3%), organochlorine pesticides (0.3%), artificial UV (0.4%) and nickel (0.8%) were the rarest. Considerably high proportions of workers were exposed to environmental tobacco smoke (23.4%), silica (16.6%), mineral oils (12.7%) and chlorinated solvents (11.1%), as well as chromium (10.1%). When the level of exposure was further classified into low, medium and high, high-level exposures were mainly seen for silica (9.1%), wood dust (5.8%) and diesel exhaust (5.8%) (Table 2).

**Figure 1:**
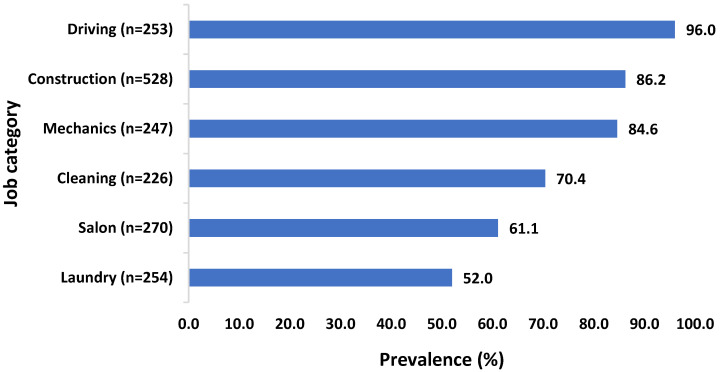
Prevalence of exposure to any carcinogens in various jobs held by migrant workers in the UAE.

Exposures to carcinogens were observed to be disproportionate among different types of jobs. The overall prevalence of exposure to any carcinogenic agents within job categories is illustrated in Figure 1. While mechanics had the highest prevalence of exposure to diesel exhaust (62.3%) and mineral oils (43.7%), laundry workers were most frequently exposed to chlorinated solvents (39.8%), including tetrachloroethylene (PERC) (36.6%) and trichloroethylene (28.3%) (Table 3). On the other hand, drivers and cleaners were mostly exposed to diesel exhaust (95.7%) and mineral oils (46.5%), respectively. About three in ten participants working in salons had probable exposure to formaldehyde (29.6%) and environmental tobacco smoke (28.9%). Construction workers were most frequently exposed to silica (48.5%), environmental tobacco smoke (33.7%), chromium (31.6%) and wood dust (27%) (Table 3).

### 3.4. Self-Rated Health

**Figure 2:**
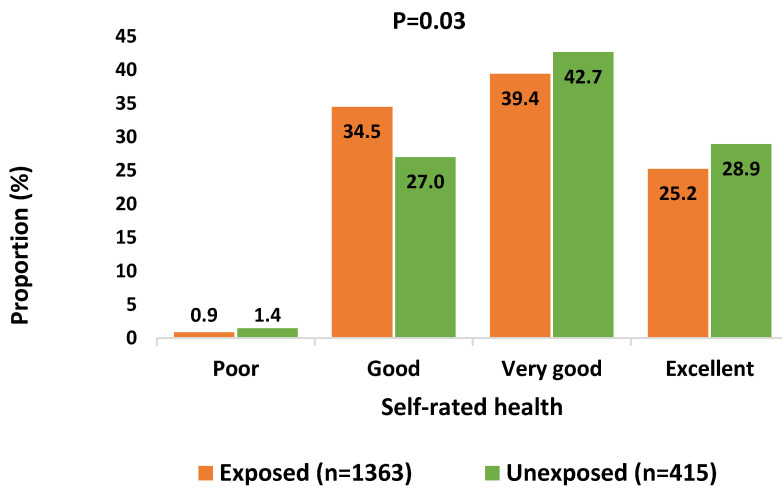
Exposure to carcinogens by self-rated health in migrant workers in the UAE.

Participants were asked to rate their overall health. Around 60% of them rated their health status as excellent (26.1%) or very good (40.2%), and only 18 participants (1.0%) as poor. There was a significant difference (*p* = 0.03) in self-rated health between workers exposed and those not exposed to carcinogens (Figure 2). Workers occupationally exposed to carcinogens had significantly lower chance to have a higher health rating in the crude analysis (COR = 0.781, 95% CI [0.638–0.957]), as well as when adjusting to potential confounders (AOR = 0.783, 95% CI [0.638–0.961]) (Table 4).

## 4. Discussion

To our best knowledge, this is the first study to attempt to estimate carcinogenic exposures among workers in the UAE. It is also the first study in the UAE to use the OccIDEAS tool for estimating exposure to carcinogens among migrant workers. Using primarily the IARC classification groups 1 and 2A, and some carcinogenic agents from group 2B, the OccIDEAS tool aims to assess exposures to any agents incorporated in the system, based on the nature of work tasks and occupational environments [3].

Few studies thus far have attempted to investigate the burden of carcinogenic exposure among working populations in the Middle East. There have been a limited number of studies examining exposure in the Arab region. The studies that have estimated certain levels of exposure were primarily conducted in Iran and Saudi Arabia among specific populations [36,37,38,39,40,41]. Agents, such as asbestos, pesticides, radon and formaldehyde, were the focus of investigation in these countries [38,39,40]. It is well established that cancer, which is the most severe health outcome related to these exposures, is one of the leading causes of mortality and morbidity in this region [42].

Twenty carcinogenic agents that workers in cleaning, dry cleaning, hair salons, driving, construction, carpentry and mechanical workshops in our study are exposed to were identified. The largest proportion of workers were exposed to diesel exhaust and environmental tobacco smoke in general. Estimated high-level exposures were observed for silica, wood dust and diesel exhaust. The pattern of exposure varied by occupation. Car and bus drivers were mostly exposed to diesel exhaust, while dry cleaners to chlorinated solvents and construction workers to silica.

A similar study conducted in Nicaragua and Panama in 2013 also reported high levels of exposure to diesel exhaust and environmental tobacco smoke among workers [43]. Additionally, the study found that solar ultraviolent radiation poses a high exposure to workers in both countries. UV radiation was not identified to pose a high risk to the participant workers of this study, a matter which could be explained by cultural reasons of wearing clothes that fully cover the body and by ongoing preventive measures introduced by the UAE Government in 2020 to reduce UV exposure among workers by implementing a noon work ban between 12.30 and 3.00 p.m. for jobs performed outdoors under the sun, from mid-June to mid-August [44]. Moreover, to ensure better workers’ safety and rights, the UAE launched a new Federal Labor Law effective since February 2022. The law aims to develop the legislative structure for different types of work, especially in the private sector [45].

Migrant workers in the UAE are sometimes employed in occupations that they may not have previous experience in or training for. Although safety regulations are introduced and enforced all over the UAE, many workers may have language barriers to fully understand and follow safety guidelines and regulations. It is well known worldwide that migrant workers have the tendency to accept demanding jobs, a phenomenon that may imply several problems, including higher exposure to carcinogens [9]. In the UAE, expatriate workers may not stay long enough in the country to experience the long-term health effects resulting from work-related exposures, such as cancer. It is imperative, therefore, to establish baseline information and then regularly monitor carcinogenic workplace exposures. Valid exposure data are absolutely essential to plan and introduce adequate mitigation measures to eliminate or reduce the adverse consequences.

Workers’ exposure to different carcinogenic agents were found to vary considerably by the type of occupation. This obvious finding was also reported by several earlier studies [17,18,19,43,46,47,48]. A recent study in Korea identified similar types of exposures among workers in similar occupations [28]. The Korean study found welding fumes to cause the highest prevalence of exposure to a large number of various groups of industrial workers, followed by ultraviolet radiation, ionizing radiation and mineral oil mist [28].

An important finding of this study is that our participants rated their health quite positively. This could be due to the fact that the study population was relatively young and physically active. Since typically healthy people migrate to work in another country and all migrant workers have to undergo a preemployment fitness-to-work medical checkup in the UAE, the healthy worker effect, which is generally observed in working populations [49], is expected to be especially strong among the UAE migrant workers. Other possible explanations include the way people perceive their health and wellbeing and its relation to the workplace [50]. Despite self-rating their health generally high, we found a statistically significant inverse correlation between workplace exposure to carcinogens and the level of health in the crude analysis as well as after adjusting for potential confounders. This observation indicates an existing association even in a cross-sectional study design. Studies have referred to self-health rating as being a predictor for mortality and working sustainability [51,52], and an association between working hours and a poor self-health rating was established [52].

### Strengths and Limitations

This is the first study conducted in the UAE and in the region to estimate occupational exposures to carcinogens by agents and by work type using the OccIDEAS survey tool. The survey was conducted in two major cities of the UAE on a relatively large sample. A major limitation of the study is its cross-sectional design. Although the study found an association between occupational carcinogenic exposure and self-rated health, causal relationship cannot be established. Information bias deriving from language barriers, recall bias and reporting bias due to concern about full disclosure of specific work circumstances and/or health conditions may have influenced the validity of self-reported data.

## 5. Conclusions

This is the first study of its kind in the UAE to investigate the current prevalence and level of exposure to carcinogens among immigrant workers. We found that migrant workers were exposed to several different carcinogens, mainly environmental tobacco smoke, diesel exhaust, asbestos and formaldehyde. The results of the study provide gap-filling information that will be of particular interest to national, regional and international organizations, policy makers and researchers in the field of occupational health and cancer studies. These data on occupational carcinogenic exposures allow for evidence-based planning and the implementation of preventive measures, as well as providing a baseline for further studies.

## Figures and Tables

**Table 1:** Demographic, lifestyle and occupational characteristics of the participants in a study of migrant workers in the UAE (N = 1778).

Demographic Characteristics	Frequency	Percentage
**Age (median, IQR)**	32	(IQR = 9)
**Gender**		
Female	146	8.2
Male	1632	91.8
**Emirates**		
Abu Dhabi/Al Ain	1057	59.4
Sharjah	721	40.6
**Language**		
English	690	38.8
Hindi	556	31.3
Urdu	439	24.7
Other	93	5.2
**Highest education**		
Did not go to school	274	15.4
Primary/Quran school	809	45.5
Middle or secondary school	477	26.8
College or University	218	12.3
**Income**		
<1500 AED	729	41
1500–3000 AED	709	39.9
>3000 AED	340	19.1
**Birth country**		
Bangladesh	342	19.2
India	552	31
Nepal	83	4.7
Pakistan	531	29.9
Philippines	85	4.8
Other	185	10.4
**Current smoker**		
No	1427	80.3
Yes	351	19.7
**Alcohol consumption**		
No	1521	85.5
Yes	257	14.5
**Company size**		
1–5 (micro)	547	30.8
6–19 (small)	759	42.7
20–199 (medium)	358	20.1
200 or more (large)	114	6.4
**Job type**		
Construction	528	29.7
Cleaning	226	12.7
Driving	253	14.2
Laundry	254	14.3
Salon	270	15.2
Mechanics	247	13.9
**Years in job**		
1–5	946	53.2
6–10	520	29.2
>10	312	17.5

**Table 2:** Prevalence of exposure to specific carcinogens by estimated exposure level among migrant workers in the UAE (N = 1778).

Occupational Carcinogen	Level of Exposure							
	Low		Medium		High		Total	
	n	%	n	%	n	%	n	%
Formaldehyde	121	6.8	45	2.5	0	0	166	9.3
Asbestos	9	0.5	46	2.6	0	0	55	3.1
Silica	92	5.2	43	2.3	161	9.1	296	16.6
Arsenic	11	0.6	6	0.3	0	0	17	0.9
Cadmium	3	0.2	1	0.1	0	0	4	0.3
Chromium VI	166	9.3	13	0.7	1	0.1	180	10.1
Lead	72	4	9	0.5	1	0.1	82	4.6
Nickel	7	0.4	6	0.3	1	0.1	14	0.8
Mineral Oils	103	5.8	122	6.9	0	0	225	12.7
Wood Dust	15	0.8	26	1.5	103	5.8	144	8.1
Organochlorines	2	0.1	4	0.2	0	0	6	0.3
Environmental Tobacco Smoke	390	21.9	27	1.5	0	0	417	23.4
Diesel Exhaust	108	6.1	399	22.4	103	5.8	610	34.3
Artificial UV	1	0.1	6	0.3	0	0	7	0.4
Ocular UV	18	1	10	0.5	3	0.2	31	1.7
Solar UV	22	1.2	32	1.8	38	2.1	92	5.1
Benzene	22	1.2	32	1.8	38	2.1	92	5.1
Chlorinated Solvents	133	7.5	24	1.3	41	2.3	198	11.1
Tetrachloroethylene PER	65	3.7	4	0.2	38	2.1	107	6
Trichloroethylene	50	2.8	3	0.2	21	1.2	74	4.2

**Table 3:** Prevalence of exposure to specific carcinogens in various jobs held by migrant workers in the UAE (N = 1778).

Carcinogen	Driving (n = 253)	Construction (n = 528)	Mechanics (n = 247)	Cleaning (n = 226)	Salon (n = 270)	Laundry (n = 254)
Formaldehyde	0	15.7	1.2	0	29.6	0
Asbestos	0	2.7	16.6	0	0	0
Silica	13.4	48.5	2.4	0	0	0
Arsenic	0	3.2	0	0	0	0
Cadmium	0	0	1.6	0	0	0
Chromium VI	0	31.6	5.3	0	0	0
Lead	0	9.5	2	0	10	0
Nickel	0	0.4	4.9	0	0	0
Mineral oils	4.7	0.4	43.7	46.5	0	0
Wood dust	0	27.3	0	0	0	0
Organochlorines	0	1.1	0	0	0	0
Environmental tobacco smoke	14.6	33.7	15.4	23.5	28.9	13
Diesel exhaust	95.7	32.6	62.3	17.7	0	0.8
Artificial UV	0	0.4	2	0	0	0
Ocular UV	2	16	1.6	2.7	0	0
Solar UV	10.3	8.7	2	6.6	0	0
Benzene	10.3	8.7	2	6.6	0	0
Chlorinated solvents	0	0.8	10.5	18.1	9.6	39.8
Tetrachloroethylene	0	0	0.8	2.2	2.6	36.6
Trichloroethylene	0	0	0	0	0.7	28.3

**Table 4:** Association between health rating and exposure to carcinogens in migrant workers in the UAE.

Predictor	N	Crude OR (95% CI) ^a^	Adjusted OR (95% CI) ^a^
**Exposure to carcinogens**			
Unexposed	415	1 (ref)	1 (ref)
Exposed	1363	0.781 (0.74–0.96)	0.783 (0.63–0.96)

^a^ Adjusted for age, sex, smoking and alcohol consumption.

## Data Availability

The data presented in this study are available on request from the corresponding author. The data are not publicly available due to privacy.
